# Common *MFRP* sequence variants are not associated with moderate to high hyperopia, isolated microphthalmia, and high myopia

**Published:** 2008-03-04

**Authors:** Ravikanth Metlapally, Yi-Ju Li, Khanh-Nhat Tran-Viet, Anuradha Bulusu, Tristan R. White, Jaclyn Ellis, Daniel Kao, Terri L. Young

**Affiliations:** 1 Duke University Eye Center, Durham, NC; 2 Duke University Center for Human Genetics, Durham, NC

## Abstract

**Purpose:**

The membrane-type frizzled-related protein (*MFRP*) gene is selectively expressed in the retinal pigment epithelium and ciliary body, and mutations of this gene cause nanophthalmos. The *MFRP* gene may not be essential for retinal function but has been hypothesized to play a role in ocular axial length regulation. The involvement of the *MFRP* gene in moderate to high hyperopic, isolated microphthalmic/anophthalmic, and high myopic patients was tested in two phases: a mutation screening/sequence variant discovery phase and a genetic association study phase.

**Methods:**

Eleven hyperopic, ten microphthalmic/anophthalmic, and seven non-syndromic high-grade myopic patients of varying ages and 11 control subjects participated in the mutation screening phase. Sixteen primer pairs were designed to amplify the 13 exons of the *MFRP* gene including intron/exon boundaries. Polymerase chain reactions were performed, and amplified products were sequenced using standard techniques. Normal and affected individual DNA sequences were compared alongside the known reference sequence (UCSC genome browser) for the *MFRP* gene. The genetic association study included 146 multiplex non-syndromic high-grade myopia families. Seventeen intragenic and flanking single nucleotide polymorphisms (SNPs) were chosen for the *MFRP* gene and genotyped in the large data set using the Taqman^®^ allelic discrimination assay. The family-based association Pedigree Disequilibrium Test (PDT) and GenoPDT were performed.

**Results:**

The average spherical refractive error of the hyperopic patient cohort was +4.21 diopters (D; range +2.00 to +9.25 D) and of the myopic patient cohort was −12.36 D (range −8.25 to −14.50 D). A total of 16 SNPs were identified by direct sequencing. No significant association was determined between the 16 *MFRP* gene SNPs and the moderate to high hyperopia, microphthalmia/anophthalmia affection status, and high myopia. Family based association analysis did not reveal any association between the 17 SNPs genotyped in the larger family data set for any refractive error type.

**Conclusions:**

Sequence variants of the *MFRP* gene do not appear to be associated with either the less severe forms of hyperopia, extreme forms of limited eye growth and development, or high myopia. These results indicate that the *MFRP* gene may not play a role in regulating ocular axial length in these phenotypes.

## Introduction

The coordinated growth-related changes of various components of the eye are required to achieve and maintain emmetropia (the ideal refractive state). During this process of regulated ocular growth, the axial length (distance along the visual axis between cornea and retina) is a critical determinant of refractive status [1,2]. Disruption in the regulation of ocular growth results in the development of refractive error. It is now widely accepted that both environmental and genetic influences contribute to refractive error development. Recently, four independent mutations were identified in the membrane-type frizzled-related protein (*MFRP*) gene associated with nanophthalmos, a rare disorder of eye development characterized by extreme hyperopia (farsighted refractive error) [3]. Mutations in the *MFRP* gene have also been reported in a distinct autosomal recessive ophthalmic syndrome characterized by microphthalmos, retinitis pigmentosa, foveoschisis, and optic disc drusen [4].

*MFRP* is a gene that is selectively expressed in the retinal pigment epithelium (RPE) and ciliary body [3] and encodes a transmembrane receptor protein that contains a cysteine-rich domain (CRD) [5]. CRDs are binding motifs for wingless-type proteins (WNTs), a family of cell signaling molecules that mediate cell growth, differentiation, and development, and along with frizzled proteins, are implicated in eye development and disease ([6] for a detailed review). While Ayala-Ramirez et al. [4] stated that the *MFRP* gene is necessary for photoreceptor maintenance due to the severe rod-cone dystrophy of their patients, Sundin et al. [3] maintained that *MFRP* is not critical for retinal function. However, Sundin et al. [3] proposed a role for *MFRP* in the regulation of ocular axial length.

High hyperopia, microphthalmia, anophthalmia, and high myopia are all ocular disorders that present with altered ocular axial length. While microphthalmia and anophthalmia with very high hyperopia are rare and extreme phenotypes [7], moderate to high hyperopia [8] and high myopia ([9] for a detailed review) are relatively more prevalent. All of these conditions with high degrees of refractive error potentially result in secondary complications and visual loss. High degrees of hyperopia are associated with a narrow angle between the iris and cornea, an expansion of the choroidal vascular bed, choroidal folds, and thickened sclera [10–12] and can lead to angle closure glaucoma and retinal detachment (RD). High myopia is frequently associated with cataract, glaucoma, retinal detachment, and posterior staphyloma with retinal degenerative changes including choroidal neovascularization [13,14]. In addition to pathology leading to visual loss, refractive error prevalence has a high public health and economic impact [15], thus, it is important to understand ocular growth regulation in refractive error development.

We hypothesized that the *MFRP* gene is important in the ocular axial length regulation of all types. In this study, we screened for sequence variants in the *MFRP* gene and tested for genetic association in moderate to high hyperopic, non-syndromic microphthalmic or anophthalmic, and non-syndromic high myopic patients. We sought to investigate whether sequence variants of *MFRP* are associated with less severe forms of hyperopia, other extreme forms of limited eye growth and development or high myopia and whether the *MFRP* gene plays a role in regulating ocular axial length in general.

## Methods

### Patient information

Informed consent was obtained from all participants, and total genomic DNA was extracted from venous blood using AutoPure LS^®^ DNA Extractor and PUREGENE™ reagents (Gentra Systems Inc., Minneapolis, MN). The study was approved by the Institutional Review Board at Duke University and followed the principles of the Declaration of Helsinki. Eleven moderate to high hyperopic subjects, 10 microphthalmic/anophthalmic patients, 11 control subjects, and seven high-grade myopic subjects of varying ages participated in the sequencing study. A large data set comprising of 146 multiplex families that included 634 subjects was used for the genotyping study. Those with systemic conditions and syndromic disorders that could predispose them to their refractive status were ruled out by an ophthalmic physician and geneticist if necessary.

### Primers

**Figure 1:**
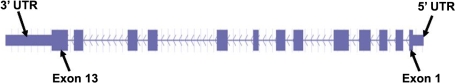
Schematic representation of the genomic arrangement of *MFRP* on chromosome 11q23.3. The vertical boxes represent exons, the horizontal bars represent introns, and the arrow heads represent the direction of transcription (source; UCSC genome browser).

The *MFRP* gene maps to chromosome 11q23.3 and spans the distance between genomic positions 118716837 and 118722580 (UCSC genome browser - Human Mar. 2006 [hg18] assembly). It is composed of 13 exons, and its genomic structure is detailed in Figure 1. Sixteen primer pairs were designed to amplify the identified 13 exons including the 5′ and 3′ untranslated regions (covering 50–150 base pairs [bp] of each intron-exon boundary; Table 1).

**Table 1:** *MFRP* gene primers for sequence variants’ screening.

**Exon**	**5′-3′ Sequence**	**Fragment size (bp)**	**Chromosomal location**
Exon1_F	CAGCTGAGTTGGATTAAGGGAC		118722259
Exon1_R	AGACTCAGGCTCGAAGGCAG	435	118722693
Exon2_F	CTTGTGCCATGAAGGACTTCTC		118722013
Exon2_R	CTGGAGAGCAGGAGGACACAG	429	118722441
Exon3_F	CAGCTCCCTGGCATGGTAAC		118721847
Exon3_R	CTCTGGAGGCGAGAAGATGG	360	118722206
Exon4_F	CTTCTCCTGGCTCTGTGTCC		118721553
Exon4_R	GCTGCAGAGATGGAGGTTAGAG	493	118722045
Exon5_F	ACAGTCCAGTGAGTACTGGGG		118721219
Exon5_R	CCCAGAGGTCTAGTCTCTCAATTTC	482	118721700
Exon6_F	AGAGCTCCCTGGGACATCAG		118720655
Exon6_R	GAACTCATCATGGGCACAGC	419	118721073
Exon7_F	GGTGAGTGTGTTCCCACCTG		118720437
Exon7_R	GACTGAGCAGGAAATGCTGAC	358	118720794
Exon8_F	CTTTGGCCCAGAACTTGTTTC		118720107
Exon8_R	CCGTCTGCTTGATCTCTGACC	327	118720433
Exon9_F	TGAAACCAGGTAAATTAAAGGTCC		118719592
Exon9_R	TTAGGGTGATGGTGAAGAGACC	447	118720038
Exon10_F	CAGACCCTAACCCTGTGTCTTC		118718595
Exon10_R	CACACCCTTACACCCTCCTG	465	118719059
Exon11_F	AATGCCACGGAGAGTAGGTG		118718370
Exon11_R	GTGAAGTGGTCCCAGAGTCAG	436	118718805
Exon12_F	ATCCTCTTGCTTCCTCACAACC		118717661
Exon12_R	AAATGCTGGTAGCAGGGCAG	374	118718034
Exon13_1_F	AGGAGGTGGTAGAGGTCCTCAG		118717426
Exon13_1_R	AAAAGAGGACGGGCAGGAAG	383	118717808
Exon13_2_F	CACCCCACTAGGCAGTGTTC		118717141
Exon13_2_R	ATCAAGGAAAAGGTCAGAAGGC	484	118717624
Exon13_3_F	GGGGAAGAGAAGTCCTCAGC		118716989
Exon13_3_R	CAGTGGCAGAGACCAAGGAC	353	118717341
Exon13_4_F	GCATCTATTCATGTGGCAGGC		118716657
Exon13_4_R	TACTCCGGACCCTCCAGTTG	466	118717122

Sixteen primer pairs were designed to amplify all 13 exons of the *MFRP* gene covering 50–150 bp of each intron-exon boundary. “F” and “R” represent the forward and reverse primers, respectively. ‘bp’ – base pairs.

### Sequencing analyses

Polymerase chain reactions (PCRs) were performed on genomic DNA using Platinum® Taq DNA polymerase (Invitrogen Corporation, Carlsbad, CA) using a touchdown protocol (available upon request). Amplicons were visualized by means of agarose gel electrophoresis and were purified with Quickstep™ 2 SOPE™ Resin (Edge BioSystems, Gaithersburg, MD). Sequencing reactions were performed using BigDye™ Terminator on ABI3730 or 3100 Genetic Analyzer (Applied Biosystems Inc., Foster City, CA). Sequences were trimmed for quality and aligned using Sequencher™ (Gene Codes, Ann Arbor, MI). Individual DNA sequences were compared alongside the known reference sequence for *MFRP* (UCSC genome browser, Representative Refseq: NM_031433).

### Genotyping

Seventeen single nucleotide polymorphisms (SNPs) were chosen to cover the *MFRP* gene and its flanking regions. We applied SNPSelector^®^ [16] to select tagging SNPs that met the following two criteria: (1) a Pearson correlation (r^2^) of at least 0.67 in the linkage disequilibrium (LD) bins and (2) a minor allele frequency of at least 5% in the Caucasian population. The SNPs that were chosen were rs2509656, rs11217240, rs11217241, rs669462, rs948413, rs948414, rs10790289, rs12294677, rs12421909, rs883247, rs883245, rs3814758, rs12577147, rs11217244, rs7932692, rs10892353, and rs7122785. A custom TaqMan^®^ allelic discrimination assay, which consisted of a mix of unlabeled PCR primers and the TaqMan^®^ minor groove binding group (MGB) probe (FAM™ and VIC^®^ dye-labeled; Applied Biosystems Inc.), was used. This assay comprised of two unlabeled PCR primers and two allele-specific probes. PCR reactions were performed with the Taqman^®^ Universal PCR Master Mix on GeneAmp^®^ PCR System 9700 (Applied Biosystems Inc.), and the ABI7900HT Fast PCR System (Applied Biosystems Inc.) was used for reading the allelic discrimination calls.

### Statistical analyses

For sequencing data, all SNPs were tested for association using the Fisher Exact Test with a significance level threshold of 0.05. For genotyping data, family-based Pedigree Disequilibrium Test (PDT) and Genotype Pedigree Disequilibrium Test (GenoPDT) were used to test for the association of markers in groups comprising of high myopes (≤5.00 D) and moderate-high hyperopes (≥+2.00 D) in comparison to control subjects (between +0.50 and −0.50 D).

## Results

For the patient samples used for direct sequencing, the average spherical refractive error of the hyperopic patient group was +4.21 diopters (D; range +2.00 to +9.25 D) and of the myopic patient group was −12.36 D (range −8.25 to −14.50 D). The refractive status findings for the hyperopic and myopic patients are listed in Table 2.

**Table 2:** Refractive status (sphere) findings of hyperopic and myopic subjects.

**Moderate-high hyperopic subjects**	**Refractive status OD (Diopters)**	**Refractive status OS (Diopters)**
1	+8.00	+9.25
2	+4.25	+4.00
3	+8.00	+9.25
4	+2.00	+2.00
5	+2.00	+2.25
6	+5.25	+3.75
7	+3.25	+2.25
8	+2.75	+3.00
9	+2.25	+3.25
10	+2.50	+2.50
11	+7.75	+3.25
**High myopic subjects**	**Refractive status OD (Diopters)**	**Refractive status OS (Diopters)**
12	−14.25	−14.5
13	−8.25	−7.875
14	−13.75	−13.625
15	−11.00	−9.875
16	−11.50	−11.375
17	−14.50	−13.50
18	−13.25	−14.00

Moderate to high hyperopic and high myopic patients were screened for MFRP sequence variants. Refractive error was measured using streak retinoscopy to the closest 0.25 diopters (D).

A total of 16 SNPs were identified by direct sequencing. A synonymous SNP (rs36015759) in exon 5 was observed in six hyperopic (five heterozygous, one substitution), six microphthalmic/anophthalmic, two high myopic, and five control subjects (all heterozygous). Five other known SNPs (rs883245 and rs883247 in the 5′ UTR, rs3814762 in exon 4, rs2510143 in exon 5, and rs948411 in intron 9) were observed in the hyperopic, microphthalmic/anophthalmic, and high-grade myopic patients as well as the control subjects (see Table 3 for details). In addition, 10 isolated, novel SNPs were identified in exons 5 (synonymous), 6 (non-synonymous), 8 (synonymous), 9 (non-synonymous), and 13 (3′ UTR) as well as in introns 8 and 11. No significant association was obtained for any SNP with any of the phenotypes studied. In addition, the family-based association study using a larger set of family data did not reveal any significant association of markers genotyped with either the hyperopic or myopic groups when compared to the control group. The results obtained from the family-based association PDT and GenoPDT analyses are listed in Table 4.

**Table 3:** Sequence variants observed in the *MFRP* gene by direct sequencing in various phenotypes studied.

***MFRP* gene**	**Location on Chromosome 11 (base pair)**	**Nucleotide change(s)**	**Amino acid change**	**dbSNP (rs#)**	**Subject phenotype**			
					**Hyperopia (n=11)**	**Microphthalmia (n=10)**	**High Myopia (n=7)**	**Controls (n=11)**
Exon 1	118722521	C/T, C->T (sub)		rs883245 (5′ UTR)	11	8	7	8
	118722464	A/G,G->A (sub)		rs883247 (5′ UTR)	11	8	7	7
Exon 4	118721714	A/G,G->A (sub)	Val->Met	rs3814762 (non-synonymous)	6	5	5	7
Exon 5	118721489	T/C,C->T (sub)		rs36015759 (synonymous)	6	6	2	5
	118721441	C/T,T->C (sub)		rs2510143 (synonymous)	11	10	6	11
Exon 6	118720829	A/G	Met->Val	Novel (non-synonymous)	1	0	0	0
Intron 8	118720229	A/C		Novel	2	0	2	1
	118720230	C/T		Novel	0	0	0	1
Exon 8	118720283	G/A		Novel	0	0	2	0
	118720256	A/G		rs35885438 (synonymous)	0	1	0	0
Exon 9	118719814	A/G	Val->Met	Novel (non-synonymous)	1	0	0	0
Intron 9	118718992	A/T		rs948411	4	3	2	2
Intron 11	118718544	A/G		Novel	1	1	0	0
Exon 11	118718539	G/T	Ser->Iso	Novel (non-synonymous)	0	0	0	1
	118718513	A/G		rs11217241 (synonymous)	0	0	1	2
Exon 13	118717345	A/G		Novel (3′ UTR)	1	0	0	0

Six novel and 7 known single nucleotide polymorphisms (SNPs) were identified with sequence variant screening. None were associated with any of the phenotypes studied. The “rs” number refers to the reference cluster number of the polymorphism.

**Table 4:** Family-based association Pedigree Disequilibrium Test (PDT) and GenoPDT analyses.

**Markers**	**Position**	**Hyperopia (>+2D) versus Normal (−0.5 to +0.5D)**		**High Myopia (≤-5D) versus Normal (−0.5 to +0.5D)**		
		**PDT**	**GenoPDT**		**PDT**	**GenoPDT**
rs2509656	118713786	1.00	1.00		0.42	0.55
rs11217240	118715126	1.00	1.00		1.00	1.00
rs11217241	118718513	0.31	0.32		0.25	0.25
rs669462	118718715	1.00	1.00		1.00	1.00
rs948413	118719981	0.41	0.41		0.34	0.27
rs948414	118720008	0.32	0.32		0.63	0.35
rs10790289	118720375	0.37	0.37		0.59	0.86
rs12294677	118721107	1.00	1.00		1.00	1.00
rs12421909	118721111	0.24	0.4		0.36	0.47
rs883247	118722464	0.18	0.48		0.14	0.42
rs883245	118722521	0.31	0.31		0.24	0.4
rs3814758	118723028	0.18	0.48		0.42	0.75
rs12577147	118723858	0.32	0.32		0.07	0.21
rs11217244	118723994	1.00	1.00		1.00	1.00
rs7932692	118725222	0.18	0.48		0.58	0.87
rs10892353	118726659	0.32	0.51		0.54	0.78
rs7122785	118727916	0.18	0.48		0.71	0.18

p-values obtained from both tests for all the markers are listed. No significant association of markers was revealed with either the hyperopic or myopic groups when compared to the control group. D–diopters.

## Discussion

There is strong evidence for a heritable effect for high hyperopia [17,18], microphthalmia [19,20], and high myopia [9]. The *MFRP* gene is predominantly expressed in the eye and at low levels in the brain [5,21], and its selective expression in the RPE makes it an ideal candidate gene for the proposed role in axial length regulation. During ocular axial length regulation, a retino-scleral signaling cascade has been postulated to explain the changes in the scleral structure and composition [22]. The RPE layer has been proposed to transmit these signals [23]. Furthermore, the potential for MFRP interactions (via the cysteine-rich domain) with WNT proteins is suggestive of a role in eye development and disease.

In this study, mutation screening of the *MFRP* gene revealed no sequence variants that would implicate this gene in the development of moderate to high hyperopia, isolated microphthalmia, isolated anophthalmia, or high-grade myopia. In addition, we presented a family-based association study of SNPs on the *MFRP* gene using a large family data set. We did not identify any significant association results for *MFRP* markers in moderate to high hyperopia and high-grade myopia phenotypes. The family-based association tests applied to this study are known to be robust against population stratification and appropriate for multiplex families such as those screened in this study [24,25].

Our results indicate that the *MFRP* gene may not be primarily involved in ocular axial length regulation. It is possible that *MFRP* performs a different role in disorders such as isolated nanophthalmos and syndromic microphthalmia (where mutations have been reported) both of which are recognized as developmental disorders unlike hyperopia and myopia. Although the microphthalmia/anophthalmia cohort in this study could be considered developmental, we did not observe any mutations in the *MFRP* gene in this cohort. However, isolated microphthalmia and anophthalmia (unlike syndromic) have been previously attributed to mutations in the retinal homeobox gene (CHX10) [26,27], which is in concurrence with the current findings, and future studies are needed to confirm this line of thought.

No mutations were observed in exons 5 and 10, which housed the previously reported *MFRP* gene mutations that resulted in a mutant protein [3,4]. However, we observed some coding synonymous (silent) single nucleotide polymorphisms (SNPs) in exon 5 in six hyperopic and six microphthalmic/anophthalmic subjects (approximately 60%) in our sequencing data. While studies on the effect of silent SNPs on protein structure are still emerging, there is a new line of thought that silent SNPs can affect in vivo protein folding and function by altering translation kinetics [28,29]. Further studies are needed to prove this; however, if this is the case, there could be a potential role for the silent SNPs in the *MFRP* gene reported in this study and future research should take these findings into account.

Another possibility is that proteins that interact with the *MFRP* gene could be involved in the regulation of ocular axial length. It is reasonable to state that regulation of eye size is a complex process and is likely to involve multiple genes. While the previously reported *MFRP* mutations in isolated nanophthalmos and syndromic microphthalmia may not directly influence axial length programming, the resultant loss of *MFRP* interaction involved in axial length regulation could lead to reduced eye size in those phenotypes.

In conclusion, sequence variants of the *MFRP* gene do not appear to be associated with either the less severe forms of hyperopia, extreme forms of limited eye growth and development, or high myopia. Our findings indicate that *MFRP* is not primarily and directly involved in ocular axial length regulation. Future studies revealing gene mutations in disease phenotypes with altered ocular axial length and the relationship of *MFRP* with these mutant genes will shed more light on the precise role of *MFRP*.
